# Change over time of the mutagenicity in the lungs of *gpt* delta transgenic mice by extract of airborne particles collected from ambient air in the Tokyo metropolitan area

**DOI:** 10.1186/s41021-018-0113-4

**Published:** 2018-11-29

**Authors:** Yasunobu Aoki, Daisuke Nakajima, Michiyo Matsumoto, Mayuko Yagishita, Michi Matsumoto, Rie Yanagisawa, Sumio Goto, Kenichi Masumura, Takehiko Nohmi

**Affiliations:** 1 National Institute for Environmental Studies, Center for Health and Environmental Risk Research, 16-2 Onogawa, Tsukuba, Ibaraki 305-8506 Japan; 2 Azabu University, School of Life and Environmental Science, Sagamihara, Japan; 3 National Institute of Health Sciences, Division of Genetics and Mutagenesis, Kawasaki-ku, Japan

**Keywords:** Air pollutant, Carinogen, Environmental mutagen, Risk assessment, Transgenic rodent assay, Urban air

## Abstract

**Background:**

Previously we found that DNA adducts were accumulated in the lungs of the rats exposed to ambient air in the Tokyo metropolitan area. To examine chronological change in in vivo mutagenicity of airborne particles, extracts produced from samples of total suspended particulates (TSP) collected from urban air in 1980, 1990, and 2010 in the Tokyo metropolitan area were intratracheally administered into the lungs of *gpt* delta mice, and differences in mutation and mutant frequency were determined by using the *gpt* assay. In vivo mutations induced by the extracts were characterized and mutation hotspots were identified by DNA sequencing of the mutated *gpt* gene.

**Results:**

Administration of the 1990 extract at a dose of 0.3 mg/animal significantly elevated total mutant frequency to 3.3-times that in vehicle control, and the in vivo mutagenicity of the extract (induced mutation frequency per milligram extract) was estimated to be 2.0- and 2.4-times higher than that of the 2010 and 1980 extract, respectively. G-to-A transition was the most common base substitution in the vehicle control mice. However, administration of the 1990 extract increased the frequency of G-to-T transversion, which is a landmark base substitution induced by oxidative stress; furthermore, when the extract was administered at a dose of 0.15 mg, the mutant and mutation frequencies of G-to-T transversion were significantly increased to frequencies comparable with those of G-to-A transition. Similar increases in the mutant and mutation frequencies of G-to-T transversion were observed after administration of the 2010 extract. Hotspots (mutation foci identified in three or more mice) of G-to-A transition mutations at nucleotides 64 and 110 were induced by the 1980, 1990, and 2010 extracts; a hotspot of G-to-T transversions at nucleotide 406 was also induced by the 2010 extract. Previously, we showed that diesel exhaust particles or their extract, as well as 1,6-dinitropyrene, administered to mice induced these hotspots of G-to-A transitions.

**Conclusions:**

The results of the present study suggested that mutagenesis induced by extracts produced from TSP collected in the Tokyo metropolitan area induced in vivo mutagenicity via the same mechanism underlying the induction of in vivo mutagenicity by components of diesel exhaust.

**Electronic supplementary material:**

The online version of this article (10.1186/s41021-018-0113-4) contains supplementary material, which is available to authorized users.

## Introduction

Air pollution is a prioritized public health issue that is yet to be resolved in urban areas in industrial countries [1]. The major air pollutants in ambient air are oxidized gases (e.g., nitrogen oxide and ozone) and airborne particles generated by automobile engines or the burning of fossil fuels (e.g., coal and petroleum), and these pollutants are associated with respiratory organ diseases such as asthma, cardiovascular disease, and lung cancer [2–6]. Air pollution has long been recognized as a localized issue, but recently it has become a global issue because transboundary air pollutants are suspected to cause diseases in humans [7–9]. Airborne particles contain various mutagens such as polycyclic aromatic hydrocarbons (PAHs), nitrated PAHs, and oxidized aromatic compounds (e.g., quinones). Some mutagenic PAHs (e.g., benzo[*a*]pyrene (BaP) and dibenz[*a,h*]anthracene (dBahA)) and their related compounds are suspected causes of lung cancer [10–12]. Although outdoor air pollution is recognized as a human carcinogen (Group I substance classified by the World Health Organization/International Agency for Research on Cancer) [13], the mechanisms for in vivo mutagenicity and carcinogenicity of airborne particles in urban air, and therefore the health risk posed by mixture of air pollutants, remain to be determined.

The mutagenicity of airborne particles collected in various countries has been examined by using in vitro bioassay systems such as the Ames test [14, 15]. In Japan, studies have shown that mutagenic airborne particles are ubiquitously present in air samples collected in large cities [16, 17]. This suggests that inhaled airborne particles may have genotoxic effects in the lungs and other respiratory organs of humans. Indeed, inhalation of tobacco smoke or coke-oven emissions has been shown to induce DNA adduct formation, micronucleus formation, and DNA strand breaks in surrogate tissues (e.g., white blood cells) in rodents [8, 18, 19]. However, few studies have examined whether polluted urban air has genotoxic effects in the lungs and respiratory organs in humans.

In 1996–1997, our research group performed an in situ study in which we exposed rats to ambient air from the Tokyo metropolitan area, and we found that DNA adducts, which may be products with PAH, were accumulated in the lungs, nasal mucosa, and livers of the rats after 4 weeks of exposure [20] . This suggests that urban air containing particulate matters has the potential to produce DNA adducts in the lungs and respiratory organs in humans, and we hypothesized that in vivo mutagenesis would be elevated in those organs.

The *gpt* delta transgenic rodent is a useful tool for evaluating the mutagenicity of environmental mutagens (for a review, see [21–24]). In the *gpt* delta system, the *Escherichia coli gpt* gene encoding guanine phosphoribosyltransferase is carried on a lambda phage shuttle vector that is integrated into the rodent genomic DNA. After *gpt* delta rodents are exposed to a mutagen, the shuttle vector is rescued from the genomic DNA as phage particles, which are then infected to host *E. coli* in and the mutated *gpt* gene is detected by the appearance of 6-thioguanine-resistant colonies. The *gpt* delta system can be used to detect large deletions of genomic DNA. Previously, we used the *gpt* delta mouse system to show that inhalation of diesel exhaust increased in vivo mutagenesis in the mouse lung [25] and testis [26], and that intratracheal administration of diesel exhaust particles or diesel-exhaust-particle extract increased mutation frequency in the lung [25].

In the present study, we examined the chronological change in in vivo mutagenesis induced by airborne particles collected in 1980–2010 for retrospective study for assessing hazard of air pollution in past 40 years, as a preliminary study to an in situ exposure study using urban air. Extracts made from TSP collected from the Tokyo metropolitan area in 1980, 1990, and 2010 were administered to the lungs of *gpt* delta mice via a single intratracheal instillation, and the in vivo mutagenicity of the extracts was determined by means of the *gpt* assay. We estimated the mutant and mutation frequencies of the induced point mutations, and found that the 1990 extract had the highest mutagenicity of the three extracts. We also determined the mutation spectrum for each of the extracts and identified mutation hotspots on the *gpt* gene. Our results presented in this report, together with the results of previous studies, suggest that oxidative stress is the underlying mechanism through which the airborne particles induced mutagenesis.

## Materials and methods

### Collection of TSP from urban air

TSP was collected by using a high-volume air sampler, as described previously [27]. Briefly, TSP was collected by using a quartz fiber filter (Pallflex 2500 QAT-UP, 8 × 10 in.; Pall Corporation Port Washington NY, USA) over a 23-h period extending from 10 am to 9 am the next day. The flow rate was set at 700 L/min and approximately 1000 m^3^ of air was sampled over the 23-h period every 6 days (about 60 times sampling through a year). Sampling was undertaken at Shirokanedai, Minato Ward, Tokyo, Japan (sampling point: former building of the National Institute of Public Health; 35.6388 N, 139.7264E) in 1980 and 1990, and at Kagurazaka, Shinjuku Ward, Tokyo, Japan (sampling point: Tokyo University of Science; 35.6994 N, 139.7414E) in 2010. The distance between the two sampling points is approximately 6 km. The amount of TSP collected was calculated by subtracting the weight of the filter before sampling from that after sampling. After sampling, the quartz fiber filter was folded in two with the sample retained on the inside of the fold, and the filter was then wrapped in aluminum foil and stored at − 80 °C.

### Extract preparation and determination of PAH concentrations

The particle-carrying portion of the quartz filter was cut equally based on the total filter area. Cutting area varies from year to year, and the area was calculated so that TSP becomes about 500 mg when all the cut samples in the year are put together. The annual cut filter samples were combined and Soxhlet extracted for 24 h with 250 mL of dichloromethane. The extract was concentrated by using a rotary evaporator (R-205, B-490, Büchi Labortechnik AG, Switzerland) set at a pressure of 39,900 Pa [27], and then dried to a solid in a vial under gentle nitrogen flow.

To prepare samples for gas chromatography–mass spectrometry, 200 μg of extract was re-dissolved in 30 mL of hexane, and then 20 ng of deuterated PAHs (ES-2528, Cambridge Isotope Laboratories, Inc., Tewksbury, MA, USA) was added as an internal standard. The re-dissolved extract was again concentrated by using a rotary evaporator set at a pressure of 23,940 Pa [28]. A small amount of the concentrated hexane solution was then added to a preconditioned silica gel cartridge (Sep-Pak Silica Plus, Waters Corp. Milford, MA, USA) and eluted with 6 mL of dichloromethane–hexane (8:2 *v*/v). An aliquot (100 μL) of n-nonane was added to the eluent and the resulting solution was concentrated to 100 μL under gentle nitrogen flow. Finally, the solution was transferred to an insert vial (C-4000-2 W, 2 mL, National Scientific Supply Co., Claremont, CA, USA) and the amounts of PAHs in the extract were determined by means of gas chromatography–mass spectrometry, as previously reported [27].

### Intratracheal administration

*gpt* delta mice carrying approximately 80 copies of lambda EG10 on each chromosome 17 in a C57BL/6 J background [21] and C57BL/6 J mice were purchased from Japan SLC (Shizuoka, Japan). Twenty milligrams of dried extract in a glass vial was wetted with 17 μL of dimethyl sulfoxide (DMSO; Sigma, St. Louis), and 3.3 mL of PBS (pH 7.4; Gibco BRL, Life Technology, Grand Island, NY) containing 0.05%(*v*/v) Tween 80 (Nacalai Tesque, Kyoto, Japan) was added to the vial. Immediately before administration, the extract was solubilized by sonication and diluted to the required concentration with the vehicle (PBS containing 0.05%(v/v) Tween 80 and 0.5%(v/v) DMSO). For analyzing in vivo mutation, the extract (0.15–0.9 mg, the amount was indicated in the text) in 100 μL of the vehicle was administered to the lungs of male *gpt* delta mice (9-weeks old, 4–6 animals per a group) via a single intratracheal instillation [25]. For histopathological examination, 2010 extract (0.15, 0.3 or 0.6 mg) in 100 μL of the vehicle was administered to the lungs of male *gpt* delta mice (9-weeks old, 2 animals per a group).

1,2-Naphthoquinone (1,2-NQ) was dissolved in DMSO at a concentration of 40 mg/mL, and diluted to 6 or 12 μg/mL in PBS containing 0.05%(*v*/v) Tween 80 and 0.1%(v/v) DMSO immediately before administration. 1,2-NQ (300 or 600 ng in 50 μL) was twice administered to the lungs of male *gpt* delta mice (9-weeks old) via intratracheal instillation with a 14-day interval between instillations; these dose of 1,2-NQ were referred from a previous report [29], in which total 948 ng of 1,2-NQ per animal (158 ng per animal every week for 6 weeks) animal was already shown to be the tolerable dose.

For the intratracheal instillation, mice were anesthetized with 4% halothane (Hoechst Japan, Tokyo, Japan) until unresponsive to a tactile stimulus. The mice were then placed on a restraining board with linen threads to hold the mouth open, and the extract was instilled into the trachea via a polyethylene tube [25]. Control mice were treated with 100 μL PBS containing 0.05% Tween 80 and 0.5% DMSO in the extract experiment, or 50 μL PBS containing 0.05% Tween 80 and 0.1% DMSO in the 1,2-NQ experiment.

Mice were euthanized 14 days after the single instillation of extract or the second instillation of 1,2-NQ. In this study, we took the same duration after the instillation as our previous study for examining in vivo mutagenesis of extract made from diesel exhaust particles [25]. For analyzing in vivo mutation, the lungs were removed, rinsed in PBS and then weighed. After frozen in liquid nitrogen, the lungs were stored at − 80 °C. For histopathological examination, the lungs were fixed in situ by perfusing with 10% formalin neutral buffer solution (Nacalai, Kyoto, Japan) according to the procedure as previously reported [30], and were store in 10% formalin neutral buffer solution at 4 °C.

### Histopathological examination of lungs

Fixed mouse left lungs were embedded in paraffin. The middle region of left lung was sliced at 1 μm, and was stained with hematoxylin and eosin (HE). The histopathologic changes were observed in a light microscope (Keyence model BZ-X710, Osaka, Japan).

### *gpt* mutation assay

The *gpt* assay was performed as described previously [31]. Briefly, DNA was extracted from lung tissue by using a RecoverEase DNA Isolation Kit (Agilent Technologies, Santa Clara, CA, USA) and lambda EG10 phages were rescued by using Transpack Packaging Extract (Agilent Technologies). *E. coli* YG6020 were infected with the rescued phages, inoculated to M9 salt plates containing chloramphenicol (Cm) and 6-thioguanine (6-TG), and then incubated for 72–90 h at 37 °C, which enabled selection of colonies harboring a plasmid carrying both the gene for chloramphenicol acetyltransferase as well as a mutated *gpt* gene. Isolates exhibiting the 6-TG-resistant phenotype were cultured overnight at 37 °C in Luria–Bertani (LB) broth containing 25 μg/mL Cm, harvested by centrifugation (7000 rpm, 10 min), and then stored at − 80 °C.

### Polymerase chain reaction and DNA sequencing of the 6-TG-resistant mutants

A 739-bp DNA fragment containing *gpt* was amplified by means of polymerase chain reaction and sequenced as described previously with slight modification [31, 32].

### *gpt*-mutant frequency

*gpt*-Mutant frequency was calculated by dividing the number of colonies growing on agar plates containing Cm and 6-TG by the number of colonies growing on agar plates containing Cm alone. Mutation frequency was defined as the frequency at which independent mutants arose in the same animal. The frequencies for each type of mutation were calculated by dividing the number of each type of mutation in each group by the total number of mutations.

The in vivo mutagenic potency in the lung was defined as induced mutation frequency [‘average mutation frequency at a dose of extract administered’ minus ‘average mutation frequency of the corresponding vehicle control’] per amount of extract (mg) administered into the lung.

### Statistical analysis

All data are expressed as mean ± SD. Differences were examined by using Student’s *t*-test; *P* < 0.05 was considered statistically significant.

## Results

### Analysis of TSP extract

To determine the in vivo mutagenicity of components of TSP in urban air, TSP was collected in Tokyo metropolitan area at Shirokanedai, Minato Ward in 1980 and 1990, and at Kagurazaka, Shinjuku Ward, Tokyo, Japan in 2010. The TSP collected in1980 and 2010 were selected as oldest and newest one, respectively, among our samples, and the TSP collected in 1990 was selected for examining the chronological change in in vivo mutagenicity at the same sampling point, Shirokanedai. From the air samplings in 1980, 1990, and 2010, 454.34, 447.00, and 484.63 mg of TSP, respectively, was subjected to dichloromethane extraction, which provided 62.87, 58.41, and 65.50 mg of dried extract, respectively (0.138, 0.131, and 0.135 mg extract/mg TSP, respectively). The concentrations of all of the PAHs in the extracts we examined were decreased from 1980 to 2010 (Table 1). Especially, concentrations of the potent mutagenic and carcinogenic PAHs, BaP and dBahA [10–12], were decreased from 1980 to 2010 (BaP: 1980, 31.8 ng/mg extract; 2010, 8.3 ng/mg extract; dBahA: 1980, 2.6 ng/mg extract; 2010, 1.6 ng/mg extract).

**Table 1:** Concentrations of polycyclic aromatic hydrocarbons contained in the three extracts

Sampling time	Concentration (ng/mg extract)									
	FLN	Pyr	BaA	Chy	BbF	BkF	BaP	dBahA	IndP	BghiP
1980	31.7	32.0	31.7	55.5	55.4	19.5	31.8	2.6	56.5	66.3
1990	20.1	17.9	16.7	31.1	32.3	9.6	15.4	2.0	26.8	27.0
2010	18.3	13.4	7.5	17.4	18.4	5.2	8.3	1.6	12.2	12.8

*FLN* fluorene, *Pyr* pyrene, *BaA* benz[*a*]anthracene, *Chy* chrysene, *BbF* benzo[*b*]fluorene, *BkF* benzo[*k*]fluorene, *BaP* benzo[*a*]pyrene, *dBahA* dibenz[*ah*]anthracene, *IndP* indeno[1,2,3-*cd*]pyrene, *BghiP* benzo[*ghi*]perylene

### In vivo mutagenicity of the TSP extracts

**Fig. 1:**
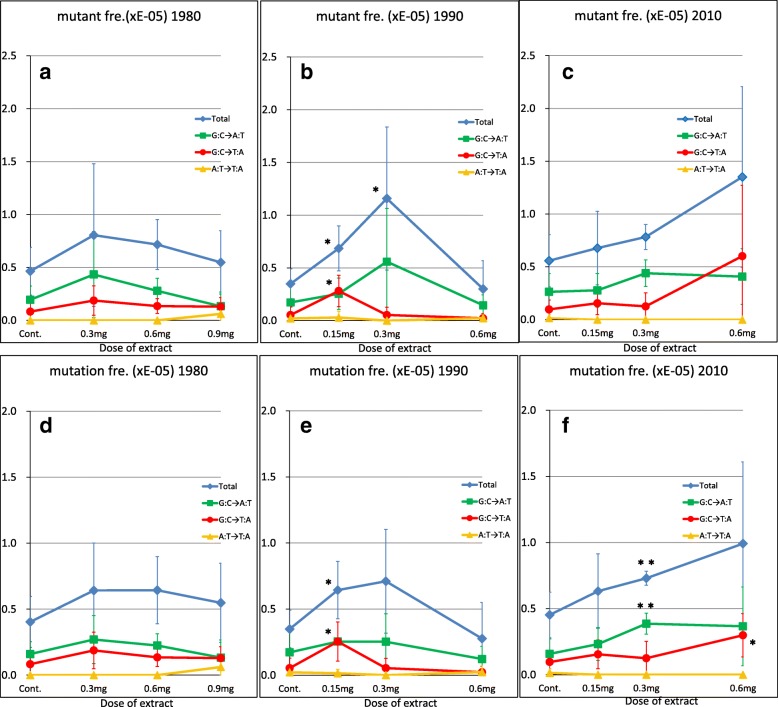
Mutant and mutation frequencies in the lungs of *gpt* delta mice administered the extracts. The data used to estimate the total mutant and mutation frequencies and the number of animals for each group were shown in Additional file 1: Table S1. The frequencies of base substitutions (G-to-A transition, G-to-T transversion, or A-to-T transversion) were calculated by dividing the number of mutants or mutations of each base substitution in an animal by the total frequency shown in Additional file 1: Table S1. Panels **a**, **b**, and **c** show the mutant frequency in mice administered the 1980, 1990, and 2010 extract, respectively. Panels **d**, **e**, and **f** show the mutation frequency in mice administered the 1980, 1990, and 2010 extract, respectively. Blue, green, red, and yellow lines show mutant/mutation frequency of total, G-to-A transition, G-to-T transversion, and A to T transversion. Symbols and bars indicate the average values and SD. *P < 0.05; **P < 0.01

To examine genomic mutations induced by the extracts, maximum 0.6 mg of 1990 or 2010 extract were intratracheally instilled to *gpt* delta mice. This dose was assumed to be adequate for detecting in vivo mutation induced by the extracts, because we previously observed significant increase in mutant frequency in the lung by intratracheal administration of 0.5 mg of diesel exhaust particles or 0.2 mg of extract made from diesel exhaust particles [25]. Maximum dose of 1980 extract was set to be 0.9 mg, since the mutagenicity of 1980 extract was expected to be lower than those of other extracts by our preliminary experiment. The mutations were identified by the *gpt* assay, and the mutant and mutation frequencies induced in the lungs of the mice by the 1980, 1990, and 2010 extracts were compared (Fig. 1 and Additional file 1: Table S1).

Administration of the 1990 extract at a dose of 0.15 and 0.3 mg (approximately 6 mg/kg body weight and 12 mg/kg body weight, respectively) significantly increased total mutant frequency to 2.0- and 3.3 times that observed in the mice administered vehicle control (0.69 × 10^− 5^ and 1.16 × 10^− 5^, respectively, vs. 0.35 × 10^− 5^; Fig. 1b). Lower total mutant frequencies were observed at higher doses in mice administered the 1980 and 1990 extracts, and this may be due to the toxicity in the lung induced by the extracts, as has been demonstrated with intratracheal administration of 1,6-dinitropyrene, which is a potent mutagen contained in diesel exhaust particles [33]. Actually, a significant decrease in the body weight by administration of 1990 extract at a dose of 0.6 mg (Additional file 2: Figure S1E) suggests that systemic toxicity was induced by this extract at the highest dose (0.6 mg). Because we estimated the average concentration of extract in the air sampled in 1990 as 0.0105 mg/m^3^, 0.3 mg of 1990 extract would be contained in 28.6 m^3^ of air, which is approximately the lifetime inhalation volume of a male mouse (assuming an average lifespan of 700 days and average inhalation volume per day of 0.04 m^3^), and this dose seemed to be adequate for assessing a life time effect of suspended particles in urban air. On the other hand, administration of 2010 elevated the mutant frequency dose-dependently, but this elevation was statistically insignificant. We measured the weight of lung at the sampling (14 days after the administration of extract). The lung weight of mice was shown to significantly increase by administration of 0.15 mg, 0.3 mg and 0.6 mg of 1990 extract (0.131 ± 0.009 g (*P* < 0.05), 0.134 ± 0.006 g (*P* < 0.01) and 0.146 ± 0.004 (*P* < 0.01), respectively) comparing to the control (0.117 ± 0.002 g) (Additional file 2: Figure S1B), and similar significant increase in the lung weight was observed by administration of 0.3 mg and 0.6 mg of 2010 extract (0.149 ± 0.010 g (*P* < 0.01) and 0.171 ± 0.006 g (*P* < 0.01), respectively vs. the control, 0.123 ± 0.010 g) (Additional file 2: Figure S1C), while the lung weight was not altered by the administration of 1980 extract (Additional file 2: Figure S1A). The relative lung weight was also significantly increased by the administration of 1990 extract and 2010 extract (Additional file 2: Figure 1H and I).

We examined histopathological changes in the lung of mice by the administration of 2010 extract (Additional file 3: Figure S2). Atypical hypertrophy and hyperplasia of bronchiolar epithelial cells, and bronchiolization were observed at bronchioalveolar junction in the lungs by administration of the extract, and were more severe at a dose of 0.6 mg (Additional file 3: Figure S2D). A marked increase of enlarged alveolar macrophages was also observed in alveoli in the lungs by administration of the extract, and inflammatory cells such as neutrophils and eosinophils were retained in capillary and interstitial tissues especially at a dose of 0.6 mg. (Additional file 3: Figure S2D and S2E), Similar histopathological changes were possibly induced in 1990 extract-administered lungs, of which weight was increased by the administration. But, edema was not shown in the lungs of extract-administered mice. Already, exacerbation of inflammation associated with increase in lung weight was shown in mouse lungs by instillation of diesel exhaust particles [34, 35]. These observations suggest that inflammation and cell proliferation were enhanced in the lungs by the administration of extract.

Because the 6-TG-resistant mutants may have included identical mutants generated via clonal expansion enhanced by cell proliferation, we also estimated total mutation frequency. The increase in total mutation frequency (i.e., the frequency at which independent mutants arose) was also significantly increased in mice administered the 1990 extract at a dose of 0.15 mg compared with that in mice administered vehicle control (0.64 × 10^− 5^ vs. 0.35 × 10^− 5^) (Fig. 1e). In mice administered the 2010 extract, both total mutant frequency and total mutation frequency were dose-dependently increased; however, only the increase in total mutation frequency induced by administration of 0.3 mg of the 2010 extract was statistically significant compared with that in mice administered vehicle control (0.73 × 10^− 5^, 1.6-times higher than vehicle control (0.45 × 10^− 5^); Fig. 1c and f).

### Types of mutation induced by the TSP extract

To determine the types of mutations induced by the extracts, we isolated 6-TG-resistant *gpt* mutants from mice administered the extracts and sequenced the mutant DNA. As shown in Table 2, G-to-A transition was the most common base substitution in mice treated with the 1980, 1990, and 2010 extracts (40% [40/100], 44% [27/61], and 39% [41/106], respectively), as well as in vehicle control mice (44% [41/93]), which is consistent with previous results from an exposure study of diesel exhaust particles in *gpt* delta mice [25] .

**Table 2:** Mutation spectra of *gpt* mutations in the lungs of *gpt* delta mice administered the extracts

Type of mutation in *gpt*	Control		1980										1990										2010									
	All (1980 + 1990 + 2010)		control		0.3 mg		0.6 mg		0.9 mg		All (0.3 + 0.6 + 0.9)		control		0.15 mg		0.3 mg		0.6 mg		All (0.15 + 0.3 + 0.6)		control		0.15 mg		0.3 mg		0.6 mg		All (0.15 + 0.3 + 0.6)	
	Number	%	Number	%	Number	%	Number	%	Number	%	Number	%	Number	%	Number	%	Number	%	Number	%	Number	%	Number	%	Number	%	Number	%	Number	%	Number	%
Base substitution																																
Transition																																
G:C → A:T	41	44	18	40	12	48	23	43	5	23	40	40	6	46	8	33	14	50	5	56	27	44	17	49	7	41	14	56	20	31	41	39
(CpG site)	(29)		(12)		(8)		(14)		(0)		(22)		(4)		(5)		(12)		(3)		(20)		(13)		(6)		(10)		(12)		(28)	
A:T → G:C	9	10	4	9	1	4	6	11	2	9	9	9	2	15	1	4	1	4	0	0	2	3	3	9	1	6	1	4	2	3	4	4
Transversion																																
G:C → T:A	17	18	9	20	7	28	9	17	6	27	22	22	2	15	9	38	2	7	1	11	12	20	6	17	4	24	5	20	26	41	35	33
G:C → C:G	1	1	0	0	1	4	2	4	1	5	4	4	0	0	1	4	1	4	1	11	3	5	1	3	0	0	0	0	1	2	1	1
A:T → T:A	2	2	0	0	0	0	0	0	2	9	2	2	1	8	2	8	0	0	1	11	3	5	1	3	0	0	0	0	0	0	0	0
A:T → C:G	6	6	4	9	1	4	3	6	0	0	4	4	0	0	1	4	2	7	0	0	3	5	2	6	0	0	0	0	1	2	1	1
Deletion																																
1	15	16	8	18	2	8	7	13	3	14	12	12	2	15	2	8	4	14	0	0	6	10	5	14	4	24	4	16	9	14	17	16
≥2	1	1	1	2	0	0	1	2	0	0	1	1	0	0	0	0	0	0	1	11	1	2	0	0	0	0	1	4	1	2	2	2
Insertion	1	1	1	2	1	4	1	2	2	9	4	4	0	0	0	0	4	14	0	0	4	7	0	0	1	6	0	0	2	3	3	3
Other	0	0	0	0	0	0	1	2	1	5	2	2	0	0	0	0	0	0	0	0	0	0	0	0	0	0	0	0	2	3	2	2
*Total*	*93*	*100*	*45*	*100*	*25*	*100*	*53*	*100*	*22*	*100*	*100*	*100*	*13*	*100*	*24*	*100*	*28*	*100*	*9*	*100*	*61*	*100*	*35*	*100*	*17*	*100*	*25*	*100*	*64*	*100*	*106*	*100*

‘All’ indicates combined data of mice treated with the different concentrations of extract. The column of ‘All (1980 + 1990 + 2010)’ indicates the sum of number of each mutation in the control for 1980, 1990 and 2010, and the percentage of each mutation

However, administration of the 1990 extract significantly increased the G-to-T transversion frequency compared with that in mice administered vehicle control (Fig. 1b and e). At a dose of 0.15 mg, both the mutant and mutation frequency of G-to-T transversion were significantly elevated to 0.28 × 10^− 5^ and 0.25 × 10^− 5^, respectively, which were values comparable to those for G-to-A transition, but the elevation of G-to-T transversion was not seen at a dose of 0.3 mg or 0.6 mg. In mice administered a dose of 0.3 mg, instead of G-to-T transversion, deletions and insertions (both 14% [4/28]) were found to contribute to the increase in total mutant and total mutation frequency (Table 2).

Administration of the 2010 extract at a dose of 0.3 mg induced a significant increase in the frequency of G-to-A transition mutations (0.39 × 10^− 5^) compared with the control (0.16 × 10^− 5^). However, at a dose of 0.6 mg, G-to-T transversion became the most common mutation (41% [26/64], Table 2), and the mutation frequency of this base substitution (0.30 × 10^− 5^) was significantly increased compared with the control (0.10 × 10^− 5^) to the same level as G-to-A transition (Fig. 1f). No significant changes were observed in the frequency of A-to-T transversion by the administration of 1990 extract or 2010 extract (Fig. 1).

G-to-T transversion is a landmark base substitution induced by oxidative stress [36, 37], which implies that the observed increases in the frequency of in vivo mutations in the lungs of the *gpt* delta mice may be driven by reactive oxygen species generated by compounds contained in the extracts.

### Mutations induced by 1,2-NQ

**Fig. 2:**
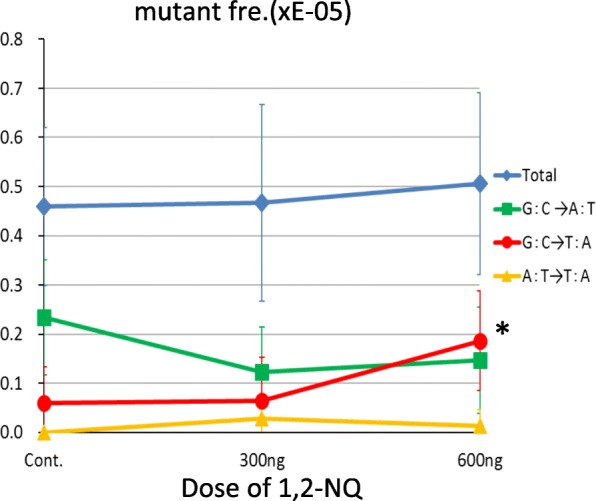
Mutant frequency in the lungs of *gpt* delta mice administered 1,2-NQ. Data used to estimate the total mutant frequencies is summarized in Additional file 4: Table S2. The frequencies of base substitutions (G-to-A transition, G-to-T transversion, or A-to-T transversion) were calculated by dividing the number of mutants in each animal (shown in Additional file 5: Table S3) by the total frequency (shown in Additional file 4: Table S2). Blue, green, red, and yellow lines show mutant frequency of total, G-to-A transition, G-to-T transversion, and A-to-T transversion. Symbols and bars indicate average values and SD. *P < 0.05

Next, we examined whether a reactive oxygen species-generating compound had the ability to induce G-to-T transversion mutations in the lungs of *gpt* delta mice, by using 1,2-NQ as a representative compound contained in diesel exhaust [29, 38]. Two-times intratracheal administration of 1,2-NQ at an interval of 2 weeks and at a dose of 300 or 600 ng did not significantly alter total mutant frequency in the mice (Fig. 2 and Additional file 4: Table S2). The most common mutation in the mice administered 600 ng 1,2-NQ was G-to-T transversion (36% [17/47]), whereas that in the vehicle control mice was G-to-A transition (47% [16/34]) (Additional file 5: Table S3). Thus, the mutant frequency of G-to-T transversion was significantly increased in mice administered 600 ng 1,2-NQ compared with that in mice administered vehicle control (0.19 × 10^− 5^ vs. 0.06 × 10^− 5^; Fig. 2), indicating that the reactive oxygen species-generating compound 1,2-NQ induced G-to-T transversion mutations in the lungs of the mice.

### Characterization of mutation hotspots on the *gpt* gene induced by the TSP extracts

**Fig. 3:**
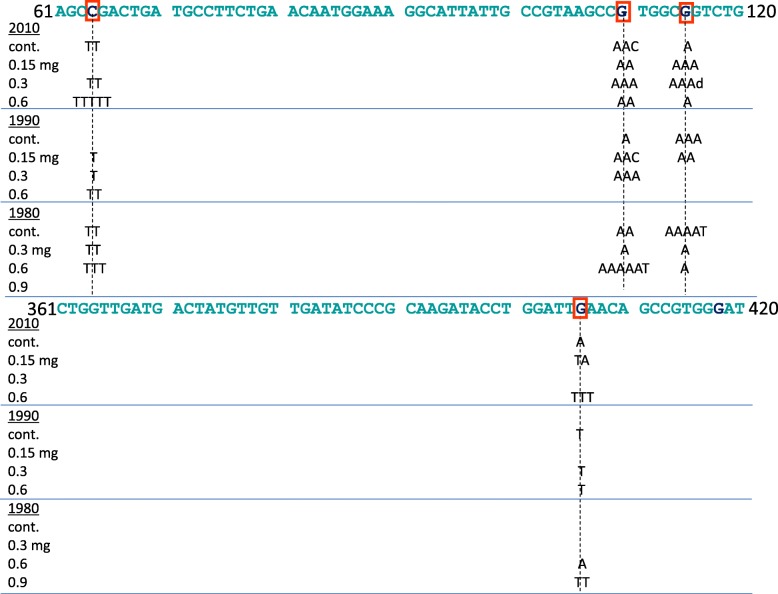
Mutation hotspots (nucleotides 64, 110, 115, and 406; red squares) on *gpt* gene in the lungs of mice treated with the 1980, 1990, and 2010 extracts. Blue letters show the *gpt* sequence from nucleotide 61 to 120 and from nucleotide 361 to 420. Dotted lines represent positions of hotspots. Black letters indicate the substituted bases, and each letter indicates independent mutations observed at the hotspots. d, deletion of base(s)

Sequencing of the 6-TG-resistant mutants revealed that administration of the extracts induced mutations at several mutation hotspots on the *gpt* gene, which we defined as mutation foci identified in three or more mice (Additional file 6: Figure S3A–C; summarized in Fig. 3) [25]. In the mice administered the 1990 extract at a dose of 0.3 mg (see Additional file 6: Figure S3B for the positions of the mutations on the *gpt* gene), nucleotide 110 was identified as a hotspot for G-to-A transition mutations. In the mice administered the 2010 extract (see Additional file 6: Figure S3C for the positions of the mutations on the *gpt* gene), nucleotides 64 (dose, 0.6 mg), 110 (dose, 0.3 mg), and 115 (doses, 0.15 and 0.3 mg) were identified as hotspots for G-to-A transition mutations. On nucleotides 64, 110, and 115, spontaneous mutations were already shown to be frequently induced [39, 40], and actually G-to-A transitions arose on these nucleotides in all control groups (except nucleotide 64 in the control for 1990 extract) in this study (Fig. 3). Since G-to-A transitions were identified on nucleotide 115 in more than three mice in the control mice (for the 1980 and 1990 extracts), we identified nucleotides 64 and 110 as hotspots induced by the extracts of TSP. These results suggest that exposure to the 1990 and 2010 extracts accelerated the induction of spontaneous mutation. Furthermore, nucleotide 406 was identified as a hotspot for G-to-T transversion mutations in the mice administered the 2010 extract at a dose of 0.6 mg (see Additional file 6: Figure S3C for the positions of the mutations on the *gpt* gene). Nucleotide 406 was not identified as a position that frequently spontaneous mutations arose, and G-to-T transition mutations were also detected at this nucleotide in mice administered the 1980 and 1990 extracts (Additional file 6: Figure S3A and S3B; summarized in Fig. 3). In the mice administered the 1980 extract, nucleotides 64 and 110 were identified as hotspots for G-to-A transition mutations at a dose of 0.6 mg.

## Discussion

Here, we report that extracts made from TSP collected within the Tokyo metropolitan area in 1980, 1990, and 2010 induced mutagenesis in the lungs of *gpt* delta mice after intratracheal instillation. This study is first report to examine the chronological change in in vivo mutagenicity of airborne particles in past 40 years using archaeological specimen, but our study has a limitation that some compounds, such as ROS-generating compounds, were possibly degraded during storage for over 30 years even if collected airborne particles were kept in − 80 °C.

The 1990 extract significantly elevated both total mutant frequency and total mutation frequency at a dose of 0.15 mg, whereas only total mutant frequency was increased at a dose of 0.3 mg (Fig. 1b and e). In contrast, administration of the 2010 extract induced an increase in total mutation frequency, but not total mutant frequency, at a dose of 0.3 mg (Fig. 1c and f). Our previous observations showed accumulation of DNA adducts in the lungs of rats inhaled to urban air [20]. The observed increases in mutation frequency reported here suggest that DNA-adduct formation induced by urban air resulted mainly in increases in base substitutions. However, since the administration of extracts induces mutations in *gpt* gene on the hotspots where spontaneous mutations arose frequently, cell proliferation accelerated by the extracts also possibly contributes to the increase in in vivo mutagenesis.

Among the three extracts, the 1990 extract was the most potent inducer of in vivo mutation in the murine lung (Table 3); the in vivo mutagenic potency (defined in the section of *gpt* mutant frequency in Materials and Methods) was estimated as 1.9 × 10^− 5^/mg extract based on average mutation frequency (0.64 × 10^− 5^) at a dose that the significant increase was shown (0.15 mg). In contrast, the in vivo mutagenic potency of the 2010 sample was estimated to be 0.93 × 10^− 5^/mg extract, based on average mutation frequency (0.73 × 10^− 5^) at a dose that the significant increase was shown (0.3 mg) lower than that of the 1990 extract. Even though significant increase in mutation frequency was not observed by administration of 1980 extract, the in vivo mutagenic potency of this extract was tentatively calculated to be 0.8 × 10^− 5^/mg lower than that of the 1990 extract based on the average mutation frequency at the dose of 0.3 mg (0.64 × 10^− 5^) (Fig. 1d). Thus, the 1990 extract had approximately 2.0-times the mutagenicity of 2010 extract, and 2.4-times higher mutagenicity than the 1980 extract.

**Table 3:** Mutagenicity and concentration of TSP in Tokyo metropolitan area in 1980, 1990 and 2010

Sampling time	In vivo mutagenic potency^a^	Concentration of TSP^b^	In vitro mutagenic potency^c^
1980	0.8 × 10^−5^	0.095	1302
1990	1.9 × 10^−5^	0.080	870
2010	0.93 × 10^−5^	0.036	

^a^*gpt* average mutation frequency per mg extract

^b^mg/m^3^

^c^His^+^ revertants per mg extract in TA98 without S9 (representative of Ames tests). Data from the reference [38]

In this study, we measured the concentration of TSP collected at the sampling points. The estimated concentration of TSP in our study decreased gradually as the sampling year increased (0.095, 0.080, and 0.059 mg/m^3^ in 1980, 1990, and 1999, respectively) at the Shirokanedai sampling point; and the concentration of TSP at the Kagurazaka sampling point in 2010 (0.036 mg/m^3^) was lower compared with Shirokanedai sampling point (Table 3). In addition, we examined the in vitro mutagenicity of extracts made from TSP collected from 1980 to 2001 by the Ames test using *Salmonella typhimurium* strains TA98 and TA100 with or without S9 mixture [41], and showed that the in vitro mutagenicity of extract assayed by TA100 (−S9) and TA98(+S9) was a similar level between 1980 extract and 1990 extract (in vitro mutagenicity in 1990: 576 and 527 revertants/mg extract; in vitro mutagenicity in 1980: 554 and 517 revertants/mg extract using TA100 (−S9) and TA98 (+S9), respectively) but that assayed by TA100 (+S9) and TA98 (−S9) was higher in 1980 extract than 1990 extract (in vitro mutagenicity in 1990: 390 and 870 revertants/mg extract; in vitro mutagenicity in 1980: 700 and 1302 revertants/mg extract using TA100 (+S9) and TA98 (−S9), respectively) (Table 3). The results described above suggest that in the present study the 1990 extract contained higher concentrations of components that induced in vivo mutations than did the 1980 extract. For example, BaP, a potent mutagen in ambient air, was expected to contribute to the in vivo mutagenicity of the 1990 extract, but the concentration of BaP in the 1990 extract (15.4 ng BaP contained in 1 mg of this extract, see Table 1) was assumed to be ignorable for in vivo mutagenicity of this extract; because the in vivo mutagenic potency of BaP was estimated to be 1.5 × 10^− 5^/mg [32], the in vivo mutagenic potency of 15.4 ng BaP was calculated to be approximately 2.3 × 10^− 10^, which was evaluated as 1.2 × 10^− 5^ part of the in vivo mutagenic potency of 1990 extract (1.9 × 10^− 5^/mg extract).

Further studies are needed to elucidate why the 1990 extract had the highest in vivo mutagenicity among the extracts examined in the present study. However, the lower in vivo mutagenicity of the 2010 extract is possibly the result of environmental protection measures implemented in the Greater Tokyo Area in the 2000s to reduce diesel engine emissions and the concentrations of TSP in urban air [42]. We revealed here chronological changes in in vivo mutagenic potency of extract made from TSP by a single intratracheal administration, however, in vivo mutagenesis induced by repeated dosing of extract also needs to be analyzed for assessing the chronic effect.

The in vivo mutagenicity of genotoxic chemicals is related to their carcinogenicity. For example, we have previously shown that the carcinogenicity of genotoxic compounds (median toxic dose [TD_50_], defined here as lifetime daily dose in mg/kg/day that induces tumorigenesis in half of the test animals) was correlated with in vivo mutagenicity (total dose [mg per kg-body weight] after administration of multiple doses of test substance per induced mutant frequency × 10^5^) [43] as a following equation.

$$ \log\ \left[\mathrm{total}\ \mathrm{dose}\ \mathrm{per}\ \left(\mathrm{induced}\ \mathrm{mutant}\ \mathrm{frequency}\times {10}^5\right)\right]=1.16719\ \mathrm{x}\ {\mathrm{logTD}}_{50}-0.023 $$

So, as a case study for adopting this correlation we derived, we used evaluate the carcinogenicity of 1990 extract from the in vivo mutagenicity, although in the present study we estimated in vivo mutagenicity after only a single intratracheal instillation of the extracts. In vivo mutagenicity (total dose of extract per induced mutant frequency × 10^5^) was estimated as 14.7 mg/kg, which was calculated from total dose (5 mg/kg body weight; dose [0.15 mg] divided by 0.03 kg [assumed average body weight of a male mouse]) and induced mutant frequency × 10^5^ (0.34) at a dose of 0.15 mg. This value corresponds to a TD_50_ value of 5.2 mg/kg/day [43]. If the body weight of a male mouse is 0.03 kg [44], then the daily intake of extract corresponding to the TD_50_ value is estimated to be 0.156 mg/day. And if the daily inhalation volume of a male mouse is 0.04 m^3^ [44], then the concentration of extract in air corresponding to the daily intake for the TD_50_ value is equal to 3.9 mg/m^3^, which is approximately 370-times the concentration of extract in the air at the sampling point in 1990 (0.0105 mg/m^3^).

Our mutation spectrum analysis showed that G-to-A transition was the most common base substitution in the lungs of the *gpt* delta mice administered the extracts (Table 2 and Fig. 1). The reason that the mutant/mutation frequency of G-to-A transition was elevated by the administration of extracts is unknown. But, decrease in mismatch repair may contribute to the increase in occurrence of G-to-A transitions in the extract-instilled mice [45]. Another possibility is that DNA cytidine deaminase, which catalyzes the conversion of cytosine to uracil [46], is activated in the lungs, causing to lead G:U mispair formation and hence mutation of G:C to A:T [47]. Administration of the extracts increased the frequency of not only G-to-A transition but also of G-to-T transversion, which is a landmark mutation induced by oxidative stress [36, 37] via formation of 8-oxo-deoxyguanine [48], and is a common mutation on TP53 gene in human lung cancer [13]. G-to-T transversion was the most common mutation in mice administered the 1990 extract at a dose of 0.15 mg or the 2010 extract at a dose of 0.6 mg, suggesting that the extract contained oxidative-stress-generating components. To confirm this possibility, 1,2-NQ (a representative generator of oxidative stress that is present in diesel exhaust and urban air [29, 38]) was instilled into the lungs of *gpt* delta mice, and the mutation spectrum was determined (Fig. 2). Although the total mutant frequency remained the same compared with control after instillation of 1,2-NQ at the doses we examined, G-to-T transversion, not G-to-A transition, was the most common base substitution in the 1.2-NQ-instilled lung at a dose of 600 ng. This suggests that oxidative stress-generating compounds, such as 1,2-NQ, increase at least in part, the mutagenicity and possibly the carcinogenicity of inhaled urban air.

It is well known that mutations are not uniformly induced on the *gpt* gene. The 1980, 1990, and 2010 extracts induced hotspots at nucleotides 64 and 110 for G-to-A transition mutations (Fig. 3). Because nucleotides 64 and 110 are part of CpG sites where spontaneous mutations are known to occur frequently [39, 40], this suggests that the in vivo mutagenicity of extracts used in the present study was partly caused by accelerated spontaneous mutation. Previously, we showed that nucleotides 64 and 110 were hotspots for G-to-A transition mutations in the lungs of mice exposed to diesel exhaust via inhalation, diesel exhaust particles via instillation, or diesel-exhaust-particle extract via instillation [25], as well as in the lungs of mice exposed to 1,6-dinitropyrene, a potent mutagen contained in diesel exhaust particles, via instillation [33], suggesting that this compound and related compounds contribute to the induction of in vivo mutations by TSP. Similarly, nucleotide 406 is a hotspot for G-to-T transversion mutations induced by the extracts examined in the present study and also by inhalation to diesel exhaust or instillation of diesel exhaust particles or their extract; however, nucleotide 406 is not part of a CpG site and G-to-T transversion at this nucleotide may have specifically been induced via oxidative stress generated by compounds contained in the sampled urban air. Together, our results indicate that exposure to TSP extract induced the same mutation hotspots as those identified in the murine lung after exposure to diesel exhaust and its components such as 1,6-dinitropyrene.

## Conclusions

Our analysis of the mutagenesis induced by extracts made from TSP collected within the Tokyo metropolitan area suggests that urban air contains compounds that induce in vivo mutations via the same mechanism that components of diesel exhaust induce in vivo mutations. In addition, the present study shows that the *gpt* delta transgenic rodent in vivo mutation assay system is a useful tool for the biomonitoring of environmental mutagens, especially when they are present together with other chemicals.

## Additional files

Additional file 1: **Table S1.** Mutant and mutation frequencies in the lungs of *gpt* delta mice administered the extracts. (XLSX 15 kb)

Additional file 2: **Figure S1.** Alterations in the body weight (A, B and C), the lung weight (D, E and F) and relative lung weight (weight of lung / body weight (× 10^− 3^) by the administration of extracts (1980 extract, A, D and G; 1990 extract B, E and H; and 2010 extract, C, F and I). Horizontal axes indicate the dose of extract (mg). *P < 0.05, **P < 0.01 and ***P < 0.001 comparing to the control. (XLSX 64 kb)

Additional file 3: **Figure S2.** H & E staining of lung tissue. (A) the lung of control mouse and the lung of mouse administered 2010 extract at a dose of 0.15 mg (B), 0.3 mg (C) and 0.6 mg (D). Arrows in panels B, C and D indicate bronchiolization at bronchioalveolar junction. (E) indicates enlarged photo of squared part in panel D. Blue, black and red arrow in panel E indicates alveolar macrophage, neutrophil and eosinophil. Scale bars; 100 μm. (PPTX 22998 kb)

Additional file 4: **Table S2.** Mutant frequency in the lungs of *gpt* delta mice administered 1,2-NQ. (XLSX 10 kb)

Additional file 5: **Table S3.** Mutation spectra of *gpt* mutations in the lungs of *gpt* delta mice administered 1,2-NQ. ‘All’ indicates combined data of mice treated with 1,2-NQ at a dose of 300 or 600 ng. (XLSX 11 kb)

Additional file 6: **Figure S3.** Positions of mutations on the *gpt* gene in the lungs of extract-treated mice and vehicle control mice. Panels A, B, and C show the mutations induced in the lungs of mice treated with the 1980, 1990, and 2010 extracts, respectively. ID is the animal identification number, which corresponds to the numbers shown in Additional file 1: Table S1. The letters A, G, T, and C indicate base substitutions, and the number of characters in brackets indicates the number of clonal mutant. Arrows indicate the position of insertion of base(s). d, deletion of base(s). (ZIP 134 kb)
