# Supplementary figures and images for: Change over time of the mutagenicity in the lungs of gpt delta transgenic mice by extract of airborne particles collected from ambient air in the Tokyo metropolitan area
Source: Genes Environ. 2018 Nov 29;40:25. doi: 10.1186/s41021-018-0113-4 (PMC6263556; doi:10.1186/s41021-018-0113-4)

## Slide 1
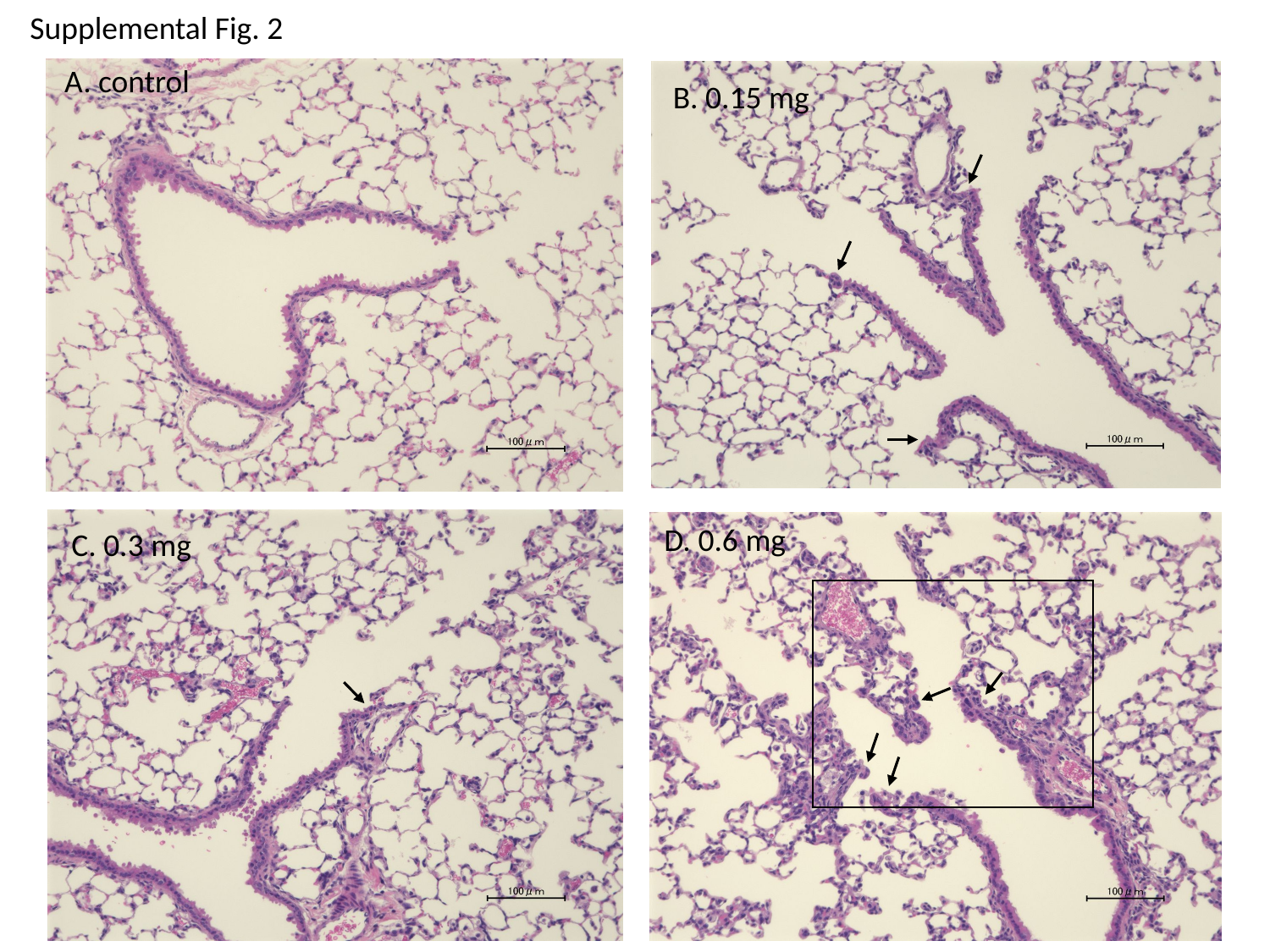

Supplemental Fig. 2
A. control
B. 0.15 mg
D. 0.6 mg
C. 0.3 mg

## Slide 2
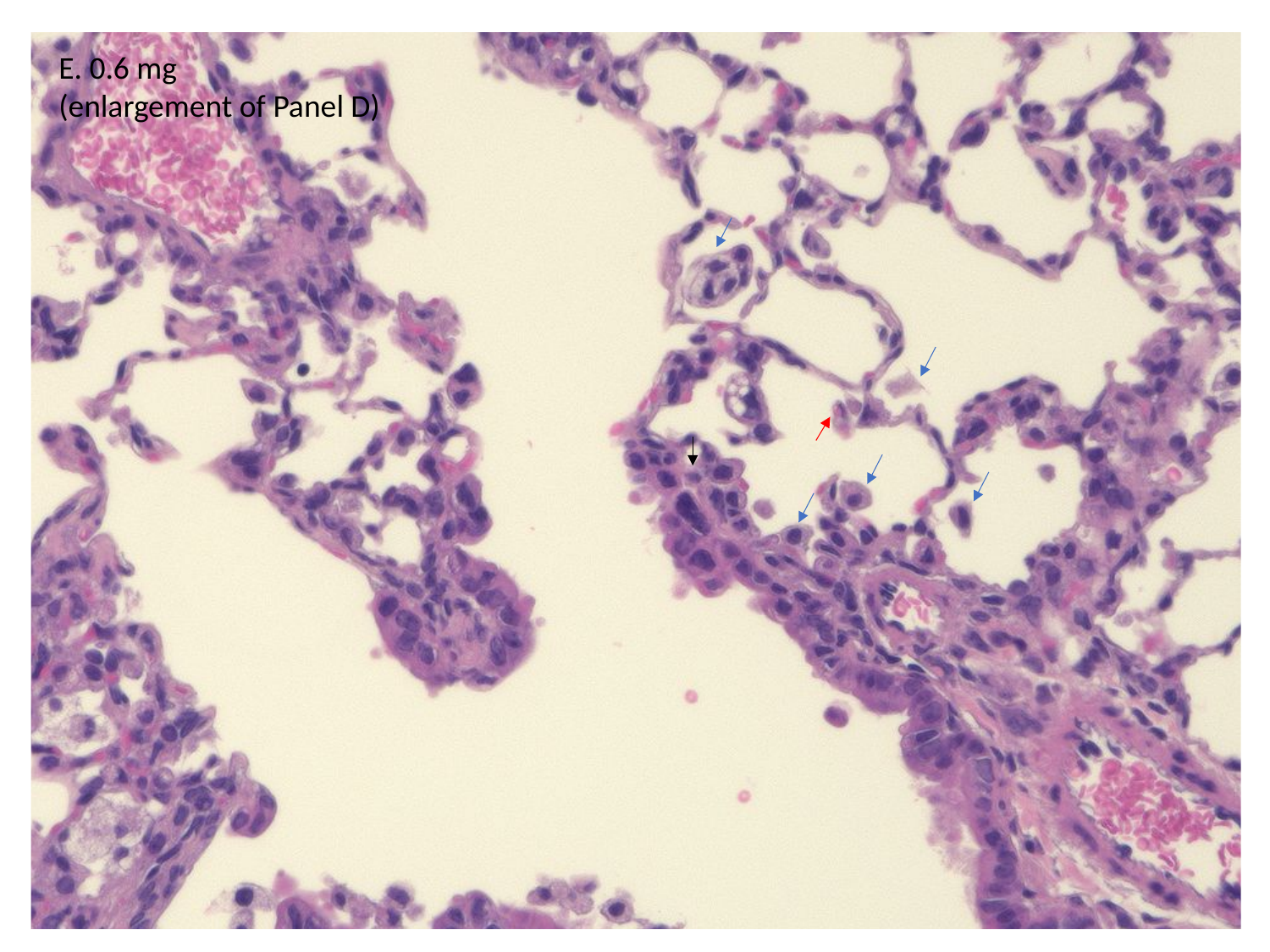

E. 0.6 mg
(enlargement of Panel D)

Supplement: Supplementary file 3 — Figure S2. H & E staining of lung tissue. (A) the lung of control mouse and the lung of mouse administered 2010 extract at a dose of 0.15 mg (B), 0.3 mg (C) and 0.6 mg (D). Arrows in panels B, C and D indicate bronchiolization at bronchioalveolar junction. (E) indicates enlarged photo of squared part in panel D. Blue, black and red arrow in panel E indicates alveolar macrophage, neutrophil and eosinophil. Scale bars; 100 μm. (PPTX 22998 kb) [file 41021_2018_113_MOESM3_ESM.pptx]
